# Association of chronic statin use, myopenia, myosteatosis and major morbidity in surgical patients with upper gastrointestinal cancer

**DOI:** 10.1007/s13304-023-01543-2

**Published:** 2023-05-18

**Authors:** Marco Cereda, Davide Paolo Bernasconi, Fabio Uggeri, Davide Ippolito, Gabriele Di Lucca, Cesare Maino, Davide Gandola, Marco Braga, Marta Sandini, Luca Gianotti

**Affiliations:** 1 HPB Unit, Department of Surgery, IRCCS San Gerardo Hospital, Via Pergolesi 33, 20900 Monza, Italy; 2 Bicocca Bioinformatics Biostatistics and Bioimaging Centre-B4, School of Medicine and Surgery, University of Milano-Bicocca, Monza, Italy; 3 School of Medicine and Surgery, University of Milano-Bicocca, Monza, Italy; 4 Diagnostic Radiology, School of Medicine and Surgery, IRCCS San Gerardo Hospital, Monza, Italy; 5 Department of Medicine, Surgery, and Neurosciences, Unit of General Surgery and Surgical Oncology, University of Siena, Siena, Italy

**Keywords:** Myopenia, Myosteatosis, Statin, Surgery, Complications

## Abstract

Derangements of body composition affect surgical outcomes. Chronic statin use may induce muscle wasting and impair muscle tissue quality. Aim of this study was to evaluate the association of chronic statin use, skeletal muscle area (SMA), myosteatosis and major postoperative morbidity. Between 2011 and 2021, patients undergoing pancreatoduodenectomy or total gastrectomy for cancer, and using statins since at least 1 year, were retrospective studied. SMA and myosteatosis were measured at CT scan. The cut-off for SMA and myosteatosis were determined using ROC curve and considering severe complications as the binary outcome. The presence of myopenia was defined when SMA was lower that the cut-off. A multivariable logistic regression was applied to assess the association between several factors and severe complications. After a matching procedure (1:1) for key baseline risk factors (ASA; age; Charlson comorbidity index; tumor site; intraoperative blood loss), a final sample of 104 patients, of which 52 treated and 52 not treated with statins, was obtained. The median age was 75 years, with an ASA score ≥ 3 in 63% of the cases. SMA (OR 5.119, 95% CI 1.053–24.865) and myosteatosis (OR 4.234, 95% CI 1.511–11.866) below the cut-off values were significantly associated with major morbidity. Statin use was predictive of major complication only in patients with preoperative myopenia (OR 5.449, 95% CI 1.054–28.158). Myopenia and myosteatosis were independently associated with an increased risk of severe complications. Statin use was associated with a higher risk of having major morbidity only in the subgroup of patients with myopenia.

**Supplementary Information:**

The online version contains supplementary material available at 10.1007/s13304-023-01543-2.

## Introduction

After complex upper gastrointestinal operations, major morbidity remains a frequent and potentially life threatening event [1]. Beside the emphasis on early identification and treatment of complications, it is of paramount importance to identify patients who are at high risk for major morbidity and to correct potentially modifiable risk factors [2].

Body composition assessment can be used for preoperative risk evaluation. Sarcopenia is a progressive decline in skeletal muscle mass, strength and performance [3], and myosteatosis is a pathologic fat accumulation in muscular tissue affecting muscle and mitochondrial function, and sustaining a proinflammatory response [4]. Myosteatosis may be associated with reduced skeletal muscle mass and acts synergistically on the decline of muscle function, but it can occur independently from muscle loss. Both conditions have been broadly associated with poor postoperative outcomes after several types of major surgical procedures for cancer [5, 6].

Statins are commonly used to prevent cardiovascular diseases by lowering blood cholesterol levels. Their mechanism of action is the competitive inhibition of the 3-hydroxy-3-methylglutaryl-(HMG)-CoA reductase, the enzyme that catalyzes conversion of HMG-CoA to mevalonate, an early rate-limiting step in cholesterol synthesis in the liver [7, 8]. Among side effects of statins, in up to 15% of treated subjects, a variety of skeletal muscle damage ranging from myalgia to severe rhabdomyolysis and statin-associated autoimmune myopathy have been described [9, 10]. Even in absence of a direct tissue damage, statin-induced myalgia may represent a limiting factor for physical activity and thus contribute to an increased risk of degenerative processes and loss of muscle mass [11, 12].

Given the potential side effects of this class of drugs on muscular integrity and given the detrimental effect of impaired muscularity on postoperative outcomes, the chronic use of statins might represent a risk factor for major morbidity after complex and challenging operations. To our knowledge, no data on the effect of statins on surgery-related outcomes have been provided so far.

Aim of this study was to evaluate the association of chronic statin use, skeletal muscle area, myosteatosis and major postoperative morbidity in surgical patients with upper gastrointestinal cancer.

## Materials and methods

### Study design and population

From prospective maintained databases at our center, patients who underwent pancreatoduodenectomy or total gastrectomy (between 2011 and 2021) for histologically proven malignancies were retrospectively analyzed.

The inclusion criteria were: the use of statins since at least 1 year, the diagnosis of a cancer of the stomach or of the pancreas suitable for upfront surgery, and the availability of a preoperative CT scan at out electronic archives. The exclusion criterion was the use of neoadjuvant chemotherapy or radio-chemotherapy.

The Charlson comorbidity index (CCI), and the American Society of Anesthesiology (ASA) score were calculated during the first outpatient visit and were used as proxy of patient frailty.

The initial cohort included 284 patients of which 70 treated and 214 not treated with statins. The final study sample was obtained through a 1:1 matching procedure. Each subject treated with statins was matched to a non-treated subject with the same profile defined by the following variables: ASA 1–2 or 3–4; age ≥ 70 vs. < 70; gender; Charlson comorbidity index < 5 vs. ≤ 5; tumor site (pancreas or stomach); intraoperative blood loss ≥ 500 ml vs. < 500.

All postoperative complications were collected and graded according to the Clavien–Dindo classification (CDC) and major morbidity was considered when the score was > 2 [13]. Specifically, the following complications were taken into account: sepsis, pneumonia, wound infection, deep abdominal collection, acute respiratory distress syndrome, delayed gastric empting, pancreatic fistula, urinary tract infection, hemorrhage, acute kidney injury. Mortality was calculated within 90 days after surgery.

The local ethical committee review of the protocol and deemed that formal approval was not required owing to the retrospective, observational, and anonymous nature of this study. The study protocol followed the ethical guidelines of the 1975 Declaration of Helsinki (revised in Brazil in 2013).

### Skeletal muscle area and myosteatosis assessments

The last available CT scan and not exceeding 30 days before surgical resection was used for the analysis. Only unenhanced scan was evaluated since muscle attenuation values measured after intravenous contrast injection are affected by tissue enhancement. All CT scans were performed with two different 256-slice scanner (iCT Brilliance, Philips Healthcare and iCT Elite, Philips Healthcare) both using a tube voltage of 120 kV and automatic tube current modulation. Raw data were reconstructed with Hybrid-Iterative Reconstruction algorithm (iDose4) if acquired on iCT Brilliance or with Iterative Model-Based algorithm (IMR) if acquired on iCT Elite. All the examinations were transferred to an image workstation (Intellispace portal 12.0, Philips Healthcare) to select and save the image in DICOM format. The analysis was performed with the open source image analysis software ImageJ (developed by the National Institutes of Health; available from http://rsbweb.nih.gov/ij/download.html), which gives comparable results of other software for body composition analysis, as previously described by van Vugt et al. [14]. The radiologists, blinded to patient information, draw regions of interest (ROIs) using standardized thresholds (− 29 Hounsfield (HU)/+ 150 HU) to obtain the cross-sectional of skeletal muscle area (SMA). The segmentation is performed on all muscles (paraspinous, psoas, abdominal) included on a single abdominal CT slice obtained at the middle level of L3 vertebra as previously described [14, 15]. The same ROIs were used to identify the intramuscular fat (myosteatosis), using specific thresholds for adipose tissue (− 190 HU/− 30 HU).

### Statistical analysis

Variables distribution was described in the overall sample and by treatment groups using median and I–III quartiles for continuous variables or absolute and relative frequencies for categorical variables. Mann–Whitney test and Chi-square test were used to compare, respectively, continuous and categorical variables between treatment groups and between groups with higher vs. lower SMA. The cut-off for SMA was determined using ROC curve methodology, specifically with the criterion of the maximum Youden index, on the non-treated group and considering severe complications as the binary outcome. The presence of myopenia was defined when the SMA was lower that the cut-off, to indicate the occurrence of clinically relevant muscle wasting. The same procedure was applied to find the optimal cut-off value for myosteatosis.

Univariate and multivariable logistic regression was applied to assess the association between several factors and severe complications. Covariates included were: statins therapy, SMA < cut-off, surgery duration, myosteatosis < cut-off. An interaction between treatment and SMA was included in order to check whether the impact of statins was different between SMA groups.

## Results

After the matching procedure, a final sample of 104 patients, of which 52 treated and 52 not treated with statins, was obtained. After 1–1 matching, the group using statins and the group no-statins were comparable for all the studied risk factors (Table 1). The median age of the population was 75 years, with an ASA score ≥ 3 in 63% of the cases. The two groups showed no significant differences in SMA and myosteatosis, while the statin-user group had marginally longer operations. Moreover, this group had also a higher proportion of postoperative morbidity than the no-statin group (71.2% vs 59.6%) and more severe complications (30.8% vs 23.1%) but without showing statistical significance (Table 1). A detailed description of the type of complications is reported in Supplementary Table 1.

**Table 1:** Descriptive statistics of the entire cohort and by treatment

Variables	Overall, *N* = 104	Statin no, *N* = 52	Statin yes, *N* = 52	*p* value*
Age, y (median [IQR])	75.0 [68.0, 80.0]	75.0 [68.0, 81.0]	75.0 [68.7, 79.2]	0.894
Age ≥ 70 years (%)	72 (69.2)	36 (69.2)	36 (69.2)	1
Sex, female (%)	40 (38.5)	20 (38.5)	20 (38.5)	1
Body mass index (median [IQR])	25.0 [22.6, 27.8]	25.7 [23.5, 28.6]	24.4 [22.4, 27.1]	0.147
ASA ≥ 3 (%)	66 (63.5)	33 (63.5)	33 (63.5)	1
Charlson comorbidity index (median [IQR])	7.0 [6.0, 8.0]	7.0 [6.0, 8.0]	7.0 [6.0, 9.0]	0.214
Charlson comorbidity index > 5 (%)	80 (76.9)	40 (76.9)	40 (76.9)	1
Skeletal muscle area, cm^2^ (median [IQR])	135.3 [108.7, 158.3]	135.5 [110.1, 162.6]	133.6 [108.1, 155.8]	0.431
Myosteatosis, HU (median [IQR])	19.5 [15.5, 27.6]	19.7 [15.3, 26.0]	19.3 [15.6, 28.7]	0.866
Type of surgery (%)				1
Total gastrectomy	48 (46.2)	24 (46.2)	24 (46.2)	
Pancreatoduodenectomy	56 (53.8)	28 (53.8)	28 (53.8)	
Duration of surgery, min (median [IQR])	292.5 [215.0, 375.0]	266.5 [219.5, 358.5]	310.0 [202.5, 381.2]	0.713
Blood loss, mL (median [IQR])	200.0 [150.0, 462.5]	200.00 [100.00, 462.50]	250.00 [150.00, 425.00]	0.748
Blood loss ≥ 500 mL (%)	26 (25.0)	13 (25.0)	13 (25.0)	1
Blood transfusion (%)	47 (45.2)	24 (46.1)	23 (44.2)	0.964
Complications (%)	68 (65.4)	31 (59.6)	37 (71.2)	0.303
Severe complications (CDC > II) (%)	28 (26.9)	12 (23.1)	16 (30.8)	0.507
Clavien-Dindo classification (%)				0.238
0	36 (34.6)	21 (40.4)	15 (28.8)	
I	8 (7.7)	2 ( 3.8)	6 (11.5)	
II	32 (30.8)	17 (32.7)	15 (28.8)	
III A	13 (12.5)	6 (11.5)	7 (13.5)	
III B	2 (1.9)	2 (3.8)	0 (0.0)	
IV A	3 (2.9)	0 (0.0)	3 (5.8)	
IV B	4 ( 3.8)	1 (1.9)	3 (5.8)	
V	6 (5.8)	3 (5.8)	3 (5.8)	

*Chi-square test for categorical variables or Mann–Whitney test for continuous variables

**Fig. 1:**
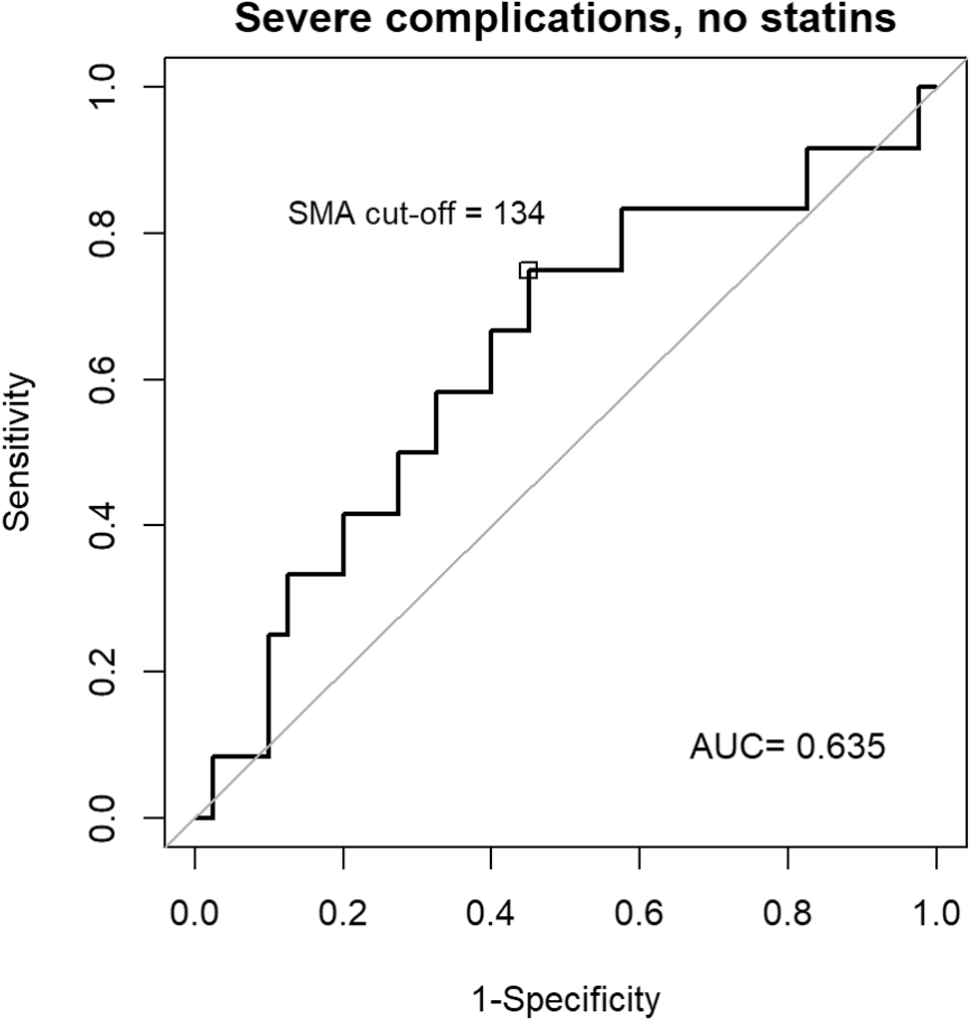
Receiver operating characteristic (ROC) curve for the cut-off of skeletal muscle area (SMA)

Figure 1 depicts the ROC curve constructed to find the optimal cut-point value of SMA associated with the onset of major complications. The area under the curve was 0.635 with an ideal cut-point of 134 cm^2^ (sensitivity = 75%, specificity = 55%; Youden Index = 0.3). The same ROC analysis was performed to find the optimal cut-off of myosteatosis. The area under the curve was 0.631 with an ideal cut-point of 26 HU (sensitivity = 50%, specificity = 82.5%; Youden Index = 0.325).

Table 2 shows the characteristics of the patients after splitting the population based on the optimal threshold of SMA. The group with SMA ≤ 134 cm^2^ (myopenia) was significantly older, had more prevalence of females, lower BMI, and longer duration of surgery. However, no significant difference on postoperative morbidity was observed. A detailed description of the type of complications according to SMA lower or higher than 134 cm^2^ is reported in Supplementary Table 2.

**Table 2:** Sample description by skeletal muscle area (SMA)

	SMA ≤ 134 cm^2^, *N* = 51	SMA > 134 cm^2^, *N* = 53	*p* value*
Age, y (median [IQR])	78.0 [72.0, 81.0]	73.0 [65.0, 78.0]	0.006
Sex, Female (%)	37 (72.5)	13 (24.5)	< 0.001
Body mass index (median [IQR])	24.4 [20.8, 26.93]	25.7 [24.1, 28.9]	0.035
Charlson comorbidity index (median [IQR])	7.0 [6.0, 8.0]	7.0 [6.0, 9.0]	0.679
ASA (%)			0.364
2	22 (43.1)	16 (30.2)	
3	28 (54.9)	35 (66.0)	
4	1 ( 2.0)	2 ( 3.8)	
Statin use (%)	26 (51)	26 (49)	> 0.99
Skeletal muscle area, cm^2^ (median [IQR])	108.7 [100.2, 121.9]	158.1 [145.1, 170.3]	< 0.001
Myosteatosis, HU (median [IQR])	19.7 [13.9, 25.2]	19.3 [16.0, 28.1]	0.507
Type of surgery (%)			0.705
Total gastrectomy	25 (49.0)	23 (43.4)	
Pancreatoduodenectomy	26 (50.1)	30 (56.6)	
Duration of surgery, min (median [IQR])	258.0 [195.0, 348.0]	320.0 [235.0, 388.0]	0.023
Blood loss, mL (median [IQR])	200.0 [100.0, 425.0]	200.0 [200.0, 500.0]	0.713
Blood transfusion (%)	21 (41.2)	22 (41.5)	> 0.99
Complications (%)	33 (64.7)	35 (66.0)	> 0.99
Severe complications (CDC > II) (%)	11 (21.6)	17 (32.1)	0.324

*Chi-square test for categorical variables or Mann–Whitney test for continuous variables

The logistic regression analysis demonstrated that patients with low skeletal muscle and myosteatosis had a significantly higher and independent risk of experiencing severe complications (Table 3). Moreover, statin use was associated with a fivefold higher risk of having major morbidity in only the subgroup of patients with SMA ≤ 134 cm^2^.

**Table 3:** Logistic regression for the risk of having severe complications with interaction between skeletal muscle area and statin use

Variables	Odds ratio (95% CI)	*p* value
Skeletal muscle area ≤ 134 cm^2^	5.119 (1.053; 24.865)	0.042
Myosteatosis ≤ 26 HU	4.234 (1.511; 11.866)	0.006
Statin yes vs no (with SMA ≤ 134 cm^2^)	5.449 (1.054; 28.158)	0.043
Statin yes vs no (with SMA > 134 cm^2^)	1.436 (0.117; 1.623)	0.216
Duration of surgery, per hour	1.293 (0.932; 1.794)	0.124

The analysis is adjusted for age, sex, and BMI

CI confidential interval, SMA skeletal muscle area

## Discussion

The present study evaluated several potential associations, namely the effect of chronic statin use on skeletal muscle mass and myosteatosis, the correlation between low muscularity and major morbidity, and the role of statins on surgical outcomes in patients undergoing operations for pancreatic and gastric cancer.

The statin-induced toxicity syndrome is gaining attention because of the drug side effects on muscle tissue. In particular, the effect of myalgia on physical activity may increase the risk of muscle wasting and impair muscle tissue quality [16]. However, studies on the association between statin use and muscle quantity and function generated contradictory results [17, 18]. Some authors, reported that in an old population, statin significantly increased the risk of sarcopenia. The consequent reduction of muscle performance was also associated with increased risk of falls, in a statin dose-dependent manner [19, 20]. Conversely, other authors evaluated statin-induced muscle toxicity and found that patients under medication were less likely to be sarcopenic [21].

The present results suggest marginal or no effects of statin use on myopenia and degeneration as measured by myosteatosis at least with the diagnostic methodology used in this study. A potential explanation lies in the different studied populations. Patients included in our study were quite old and suffered from oncologic disorders, universally recognized as inducers of muscle wasting and functional degeneration because of their intrinsic protein-wasting and proinflammatory activity [22, 23]. Therefore, it is conceivable that the effect of statins on muscular tissue has been somehow shaded by the metabolic consequences of malignant neoplasms and old age on patient metabolism and protein turnover.

Despite no differences on the SMA and myosteatosis between non- and statin users, and no direct effect of statin use on the occurrence of complications, our multivariate analysis showed that the association between chronic statins use and myopenia was independently associated with the occurrence of major morbidity. Specifically, statin users with SMA less than 134 cm^2^, experienced a fivefold higher risk of major postoperative complications.

Several hypotheses can be proposed for these findings. The first is that some patients are more sensitive to statins and they present at surgery with low muscle mass due to drug-related side effects. Another potential and more conceivable explanation is that statin side effects are more relevant in patients already suffering from low muscularity and the independent association of drug use and muscle wasting becomes detrimental on the ability of the host to recover from surgical injury.

The association of myosteatosis and adverse outcomes after major surgery has been repeatedly reported [4, 6, 24] and the present results fully confirm those previous findings with a fourfold increase of the risk of major morbidity. The pathological mechanisms of myosteatosis and its association with poor postoperative outcomes remain unclear. Myosteatosis, as well as sarcopenia may be an indicator of an individual reduced capacity to endure with physical activity [25]. This condition may match with lower cardiorespiratory performance, failure to overcome tissue injury, and inappropriate wound healing potentially increasing the risk of postoperative complications [26].

Beyond increasing health care costs and the exposing patients to failure to rescue, severe postoperative morbidity has critical implications in cancer surgery including increased risk disease recurrence and impaired tolerance to adjuvant therapies [2, 27]. Therefore, preoperative assessments to determine individual complication risk are warranted. In particular, the identification of risk factors, which can be targeted with prophylactic strategies, holds encouraging potential to improve treatment outcomes after surgical resection of gastrointestinal tumors [4],

Among the promising strategies, prehabilitation programs are gaining gradual consensus. Prehabilitation defines a program that includes a series of preadmission interventions to be initiated 3–6 weeks before surgery, aiming at improving body composition and physical performance through supervised physical activity and nutritional counselling and support. Recent studies suggest that these programs are associated with a reduced risk of morbidity after major surgical procedures [28, 29].

Decline of skeletal muscle quantity and quality is considered to be physiological with age and is one of the most important causes of functional decline and frailty in the elderly.

A substantial proportion of patients experienced postoperative morbidity and the rate of major complications was close to 27%. This picture may seem excessive if compared with other series [4, 6, 24, 30–32]. However, our study population had several baseline features of frailty. In fact, the median age was 75 years, and 70% of the patients was aged more than 70 years. Moreover, the ASA score was 3 or more in 63% of the cases, and the median CCI was 7, with 75% of the subjects having a score greater than 5.

Several study limitations should be acknowledged. First, this was a single-center retrospective study and results need to be validated by prospective observational trials. Moreover, some potentially relevant risk factors might have been missed in the retrospective data collection. Second, we used internal cut-point values for myopenia and myosteatosis rather than validated thresholds to define groups at risk. Yet, body composition may have profound regional and ethnic variations, and peculiar diseases may have different influences on anthropometric changes, emphasizing the risk of using universal thresholds. Third, no measure of muscle function was investigated, even if myosteatosis at CT scan may be considered as a proxy of muscle strength [33]. Lastly, there was a relatively small number of patients after the matching process, with a potential generation of a type-II error.

## Conclusions

In this cohort of surgical patients baring cancer of the stomach and pancreas, the chronic use of statins did not appear to affect muscle mass and quality. However, statin use was associated with a higher risk of having major morbidity but only in the subgroup of patients with skeletal muscle mass lower than the optimal threshold. Moreover, low skeletal muscle mass and myosteatosis were independently associated with an increased risk of severe complications.

### Supplementary Information

Below is the link to the electronic supplementary material.

Supplementary file1 (DOCX 144 KB)
