# Supplementary material for: Association of chronic statin use, myopenia, myosteatosis and major morbidity in surgical patients with upper gastrointestinal cancer
Source: Updates Surg. 2023 May 18;75(8):2297–303. doi: 10.1007/s13304-023-01543-2 (PMC10710384; doi:10.1007/s13304-023-01543-2)
Supplement: Supplementary file 1 — Supplementary file1 (DOCX 144 KB) [file 13304_2023_1543_MOESM1_ESM.docx]

**Supplementary table 1.** Details of complications by statin use

|  | **Statin no** | **Statin yes** | **p-value*** |
| --- | --- | --- | --- |
| Sepsis (%) | 3 ( 5.8) | 7 (13.5) | 0.318 |
| Pneumonia (%) | 10 (19.2) | 8 (15.4) | 0.795 |
| Postoperative intensive care (%) | 1 ( 1.9) | 5 ( 9.6) | 0.207 |
| Wound infection (%) | 5 ( 9.6) | 4 ( 7.7) | >0.99 |
| Deep abdominal collection (%) | 14 (26.9) | 11 (21.2) | 0.646 |
| Acute respiratory distress syndrome (%) | 2 ( 3.8) | 4 ( 7.7) | 0.674 |
| Delayed gastric emptying (grade B and C) (%) | 3 ( 5.8) | 5 ( 9.6) | 0.713 |
| Pancreatic fistula (grade B and C) (%) | 5 ( 9.6) | 8 (15.4) | 0.553 |
| Urinary tract infection (%) | 1 ( 1.9) | 1 ( 1.9) | 1 |
| Hemorrhage (grade B and C) (%) | 2 ( 3.8) | 1 ( 1.9) | >0.99 |
| Acute kidney injury (%) | 0 ( 0.0) | 2 ( 3.8) | 0.475 |

* Chi-square test

**Supplementary table 2.** Details of complications by skeletal muscle area (SMA)

|  | **SMA ≤ 134 cm^2^** | **SMA > 134 cm^2^** | **p-value*** |
| --- | --- | --- | --- |
| Sepsis (%) | 7 (13.7) | 3 ( 5.7) | 0.288 |
| Pneumonia (%) | 10 (19.6) | 8 (15.1) | 0.727 |
| Postoperative intensive care (%) | 3 ( 5.9) | 3 ( 5.7) | >0.99 |
| Wound infection (%) | 5 ( 9.8) | 4 ( 7.5) | 0.952 |
| Deep abdominal collection (%) | 10 (19.6) | 15 (28.3) | 0.419 |
| Acute respiratory distress syndrome (%) | 3 ( 5.9) | 3 ( 5.7) | >.099 |
| Delayed gastric emptying (grade B and C) (%) | 5 ( 9.8) | 3 ( 5.7) | 0.671 |
| Pancreatic fistula (grade B and C) (%) | 3 ( 5.9) | 10 (18.9) | 0.088 |
| Urinary tract infection (%) | 2 ( 3.9) | 0 ( 0.0) | 0.458 |
| Hemorrhage (grade B and C)(%) | 1 ( 2.0) | 2 ( 3.8) | >0.99 |
| Acute kidney injury (%) | 1 ( 2.0) | 1 ( 1.9) | >0.99 |

* Chi-square test
